# The stuff of memories: Planning hindsight in animal cryobanks

**DOI:** 10.1177/03063127241252081

**Published:** 2024-05-15

**Authors:** Veit Braun

**Affiliations:** University of Frankfurt, Frankfurt, Germany

**Keywords:** biobanks, cryopreservation, memory, zoology, laboratory ethnography, CryoArks, natural history museums, planned hindsight

## Abstract

Biobanks are becoming ubiquitous infrastructures in zoology and other non-human life sciences. They promise to store frozen research samples for the long term for future use. That use remains speculative but nevertheless needs to be anticipated. Following the establishment of a physical and digital infrastructure for frozen samples in an animal biobanking project, this article explores how the future is anticipated to remember the past, and how frozen objects are shaped accordingly. Situating the biobank between mundane freezing routines in a research lab and the ‘dry’ and ‘wet’ collections of natural history museums, I argue that frozen research objects need to be conserved in two separate ways. The unavailability of cryo-objects in cold storage forces researchers to store materials independently of metadata, while retaining a link between them that allows for their reunion after thawing. The result is a split object, leading a double life at sub-zero and room temperature, linked only through the surface of special plastic containers. Following the making of such split objects, this article offers an elaboration of Radin’s ‘planned hindsight’ as well as a reflection on the universality and particularity of biobanks as standardized scientific memory.

## Introduction: The persistence of memory

Deep in the belly of the Natural History Museum (NHM) in London—or slightly below ground level, to be precise—removed from the gaze of the thousands of visitors swarming its exhibition halls every day, lies a curious collection. In rooms filled with industrial freezers and shiny steel tanks, the NHM stores cells, tissue samples, DNA and other material at sub-zero temperatures. The contrast between the museum’s ‘molecular collection’ and its exhibition is stark. Metal replaces the glass and wood of the cabinets found in the museum frontstage; other than animal pictures stuck to the freezers, nothing is reminiscent of biodiversity here.

The NHM is not an exception. Throughout the world, natural history museums have become home to biobanks (Van Allen, 2017, 2018; Wrigley, 2021). Freezing biological samples at −80 (in industrial freezers) or even −196°C (in liquid nitrogen tanks) is increasingly becoming a standard practice in institutions that for the longest time have conserved their specimens ‘dry’, that is, as taxidermy, or ‘wet’, immersed in alcohol or formalin, rather than cold. In part, this development is owed to the advancement of molecular technologies whose efficiency relies on the quality of the material they are used on. While DNA, proteins and cells will fragment or denature under dry and wet preservation, their decay is greatly slowed at ultra-cool temperatures. The requirements of existing molecular techniques are only one factor behind the establishment of museum biobanks, however. Equally, if not more important, are the possibilities opened by future technologies. Experience has taught museum curators that research methods deemed flawed or outright impossible 30 years ago have become routine today (Radin, 2015). Where institutions were wise or lucky enough to retain a collection of material suitable for these technologies, their researchers can now address questions and find answers that were out of reach not long ago.

The lesson natural history museums and other research facilities have drawn from this is to bulk up their cold collections to be prepared for the coming of future technologies: The museum of the future will be one with a frozen collection that is as extensive as its other ones. Radin (2015) describes this drive to establish biobanks in zoological research as ‘planned hindsight’: Researchers might not know what their samples and research objects might be good for in the future, but they are certain that they will be more valuable tomorrow than they are today.^1^ The anticipated advance of scientific and technological progress therefore makes biobanks both a necessity and a safe bet for institutions.

In the logic of planned hindsight that Radin describes, freezers and nitrogen tanks stabilize the materiality of a preserved bio-object to allow for its semantic expansion in the future. Once thawed, the suspended samples resume their biological functions and properties. This is what allows molecular and cellular technologies to draw information from or to bestow novel potentials onto them, and enables the sequencing techniques of tomorrow to act on the materials of yesterday. Such an understanding follows a popular narrative that portrays cryotechnologies as conservative, as opposed to the transformative capacities of biotechnologies like genetic engineering, cloning, or stem cell induction. In doing so, it treats the freezer as a perfectly neutral black box: It assumes that the things that enter and leave it are indeed the same.

In this article, I question this idea of freezing as simple preservation. There is, as I will show, a lot of transformative work needed to make things fit into a freezer or a tank; in fact, biological samples never fully fit. Tissues, cells and molecules have to be split before they go into cold storage, and what remains—data on provenance, descriptors or their location in space and time—needs to be stored elsewhere, under different conditions. To be of value in the future, both parts of a ‘cryo-fact’ (Friedrich, 2020) will have to be conserved. Yet this is still not enough: What is of crucial importance is to link the cold and the warm parts again, an operation only feasible through the specific materiality of the biobank’s infrastructure. Cryopreservation poses a challenge for the preservation of memory, tied to its particular form of storage. Owing to the formatting and fixing of objects in the biobank, memories need to fit into a standardized form, which in turn dictates what can be remembered in the freezer and what needs to be recollected elsewhere. Far from being a neutral technology that merely stabilizes, conserves, and reactivates its objects, cryopreservation has to transform them first.

The empirical material for this article comes from an ethnographic study of two animal biobanking networks in the United Kingdom and several interviews with biobanking professionals and affiliated researchers, conducted in 2019 through 2022.

## Biobanks as memory devices: The case of CryoArks

CryoArks was launched in 2018 as a joint project of universities, zoos and museums. Funded by the UK’s Biotechnology and Biological Sciences Research Council (BBSRC) and until recently headed by the late Michael W Bruford, Professor of Genetics and Conservation at Cardiff University (Breithoff & Harrison, 2020), the project has built a UK-wide biobank for any animal material from wild individuals, zoo animals and rare breeds (the only exception being laboratory animals) over the last years. The aim is to make animal cells, tissue, DNA and other samples available for research by putting information on a public database and offering a platform for the physical exchange of such material. The Natural History Museum (NHM) in London, one of the consortium partners, houses part of the physical collection in the freezers and nitrogen tanks of its molecular collections facility. Further storage facilities exist at Edinburgh Zoo and the National Museums of Scotland (NMS), while Bruford’s lab at Cardiff University is in charge of coordinating the project and hosting the database.

Whereas cryopreservation has received attention from science and technology studies (STS) scholars working on animal (and plant) biobanks as an essential element in species resurrection projects (Breithoff & Harrison, 2020; Chrulew, 2017; Van Allen, 2019), CryoArks’ frozen collections are not aimed at the external world, that is, the release of resurrected species into the environment or the banking of options for a future biocapitalism. From the consortium’s point of view, they rather serve as an inward-facing infrastructure for universities, museums, zoos, and similar institutions researching animals.^2^ Nor are these collections solely future-oriented banks: in opening access to past collections and samples to researchers, they also double as archives (Peres, 2016; see also Waterton, 2010). The project aims to put the increasing amounts of zoological samples that are left behind by projects and researchers to better use—an accumulation mirrored by fewer ‘fresh’ samples, due to the disappearance of populations or legal restrictions in sample circulation after the Nagoya Protocol on access to genetic resources (Dutfield, 2017; Relly, 2023). To make the most of existing samples and to limit the need for redundant or expensive new collection efforts, CryoArks wants to provide a public repository for animal samples in that are no longer needed by their original collectors but might be of value elsewhere in the United Kingdom and abroad. At the same time, it is meant to prevent redundant collection of samples that might already exist in a laboratory elsewhere in the country. As an embodiment of a long-standing philosophy of ‘open access’ to samples under transparent conditions in biobanking (Radin, 2015), CryoArks might be understood through the economic metaphor of the clearinghouse (Millo et al., 2005), rather than that of the bank.

Yet another way to understand the planned effect of CryoArks is to view them as memory devices for scientific practice. Following Bowker’s (2008) work, ‘memory’ (the capacity to recall the past) and ‘memories’ (data from the past) should not be understood as a purely human or mental ability here. Like Peres (2016), in her study of plant seed banks as archives, I want to highlight a more general ability of institutions to relate to the past in using these terms. In many ways, this is the idea behind frozen repositories: In the ideal case, these collections should be able to function without relying on a particular person and her embodied memories. Bowker explores at length how the problem of memory has haunted scientific communities, from the beginning of geology to biodiversity collections to the databases of the 21^st^ century. Since scientific memory cannot rely on human individuals alone, objects, along with written archival sources, play an important role for remembering and re-enacting the past, as Daston (2004) has shown for type specimens as authoritative objects in remembering the particular meaning of descriptions and taxonomies by naturalists long dead. To an extent, the same principles apply to all ‘sciences of the archive’, as Daston (2012) calls them. What they differ in, nevertheless, is their conception of which memories need to be stored, which in turn can be disposed of (because they are redundant or can easily be reproduced) and, of course, the state in which a memory is best conserved. As Daston (2012, p. 162) remarks, ‘[w]hat distinguishes the sciences of the archive from other sciences … is practices of collection, collation, and preservation conceived as an intrinsically collective undertaking—and one that extends into both past and future’.

In Radin’s (2015) words, biobanking amounts to ‘planned hindsight’: Collection activities in the present are justified by pointing to future research value of frozen samples, even if no one can predict how or which samples will be of value in ten, twenty, or forty years’ time. Nevertheless, looking back from the present into the past, such efforts are validated by the tangible benefits derived from these memory infrastructures. The best example for this effect is Kurt Bernirschke’s ‘Frozen Zoo’ in San Diego, which started as a frozen collection of veterinary material Bernirschke deemed of value for descriptive cytological and genetic studies (Benirschke, 1984; Friese, 2013, pp. 108–110), much like present-day CryoArks. Only decades later, after the emergence of new molecular and cellular technologies, did it become the basis for cloning and de-extinction programs at the San Diego Zoo. This later success validated Bernirschke’s undertaking in the past and as such seems to affirm an understanding of biobanks as fueled by speculation about the future, rather than present value (Aarden, 2023; Bach & Kroløkke, 2020; Lemke 2023; Radin, 2013). Useful as it is for explaining the ongoing drive toward biobanking in the life sciences, this perspective offers more to the historian looking into the past than to the sociologist who has no access to the future other than the more or less fanciful speculations about what frozen samples might one day become.

The ‘speculation’ hypothesis also tends to focus on human imaginaries of the future, which are relatively easily retrieved through interviews and written memories, even though biobanks like CryoArks are largely meant to make the human individual redundant. The approach is thus always at a double risk: on the one hand, of slipping into a ‘sociologization’ whose access to the future relies on human imaginations and projections; and on the other hand, of ‘naturalizing’ frozen samples as biologically stable and technologically docile, always ready to serve as a screen for these speculative accounts. How exactly the former will eventually be connected to the latter, however, remains unclear. What is implied is that the future need not come at all as long as samples stand still and imagination runs wild in the present. As a consequence, it is tempting to gloss over the ways in which value is made and past, present and future are connected with each other. Looking at the activities of CryoArks in the ‘here and now’, I would like to highlight how biobanked samples, cryogenic devices, and information infrastructures, not just human imagination alone, anticipate the future and connect it with the present. The aim is to turn Radin’s ‘planned hindsight’ into a more fine-grained and specific social process: How do animal biobanks manage to square transformation and preservation? What logic is inscribed in their technological mode of recalling and recollecting? What can and cannot be remembered by animal biobanks?

## Memories material and immaterial: Organizing samples in the lab

Mafalda is always short of time. But between juggling her duties in the CryoArks project, her work as a student supervisor and her own research on population genetics, she has agreed to show me her research samples this morning. I follow her to one of about a dozen freezers in the lab room. ‘Okay, so these are the blood samples …’ The freezer is filled with boxes and cassettes full of dark red plastic tubes. Ice crystals cover most of its contents, the shelves and the freezer walls. ‘What is this …? No, this is not it. I wanted to show you the samples …’. Mafalda pulls out one box after another, shaking her head. I do not quite understand what she is looking for, but if she does not know what it looks like, at least she knows what it does *not* look like. Finally, she presents a crate of neatly labeled tubes: ‘Well, these are trout samples. But just to show you what it *should* look like. Isa-Rita’s documentation is always perfect’. She shoves the crate back into the freezer and closes the door.

**Figure 1.**
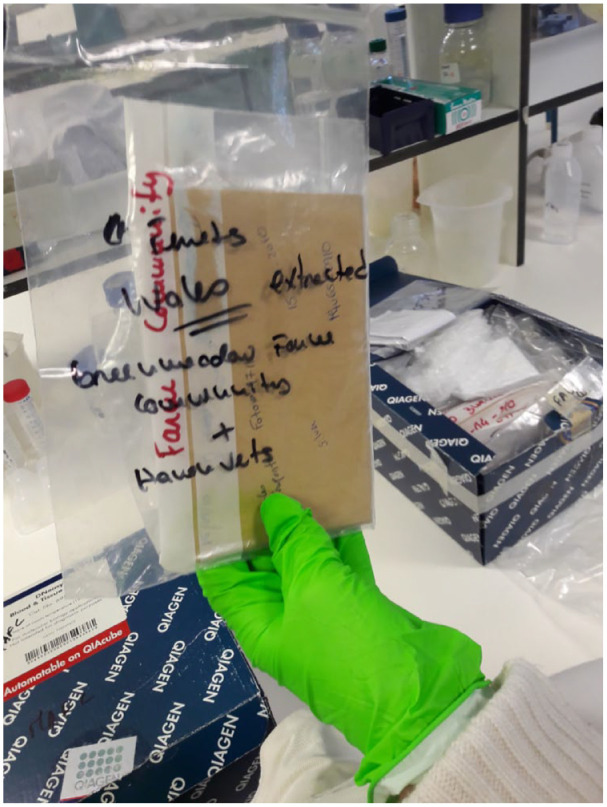
Ferret hair samples stored in envelopes.

From one of the cupboards under a lab bench, she produces a couple of cardboard boxes filled with plastic bags, which in turn contain other bags and envelopes—‘a matryoshka system of zip locks’, as Mafalda calls it (Figure 1). Rummaging through the boxes and deciphering the handwriting on the bags and envelopes, she tries to explain their contents to me. She opens one of the envelopes, pulling out a tuft of loose hairs. ‘So these are hair samples taken from domestic ferrets …’. She checks the handwriting again. ‘No, sorry, these particular ones are from wild polecats’. The samples, part of her work on polecat and ferret genetics (most of them from the UK and Portugal), were collected years ago. They have been sitting in the cupboard for a while now and Mafalda wonders if they might still be of any use for research after all this time. She has had her hands full ever since she finished her PhD, so the research on the samples is not fully finished (it never is). The hair is probably too old and dry, Mafalda concludes, but who knows, with the right technique and enough patience you probably still could extract some DNA from it. Anyway, the bigger issue is probably the bags and envelopes. ‘See? *I* always did it properly! Sex, date, location, species, breeder, fur color. But this one here …’ She shows me an envelope and sighs. ‘The person that collected these samples didn’t even write down the location. And it just says ‘June’. June of which year?’

An entire wall of the Cardiff laboratory is filled with freezers set to −20°C, each of them carrying labels that indicate their contents and their users.^3^ To the extent that this is possible, the lab is a well-organized, highly curated space. Nevertheless, retrieving scientific memories in the form of samples is more difficult than one might think. Mafalda is not entirely content with the state of things: ‘It shouldn’t look like this’, she apologizes—but of course it always does, in every lab. Labels are hastily written in cryptic handwriting, samples are moved around in need of space, people rely on their memory and are soon surprised to discover that it is not that reliable after all. Things become more complicated with more people using the lab, as there are several, often incommensurable, ways of documenting and remembering samples. At times there are four or more Catherines in the research group, so first names are not sufficient to identify the owners of samples. To make things worse, there is a constant coming and going: People finish their PhD and leave, interns and project students show up and disappear a few months later, people switch to other labs and leave behind their data but take their documentation with them. And then of course, there is the sheer diversity of samples, each demanding a different manner of description and documentation: hair, blood, feces, tissue, DNA, from invertebrates to primates.

All of this makes it very difficult to keep track of who owns what in the freezers, what is contained in a sample and what state it is in. Certainly, if everyone were as diligent as Isa-Rita in documenting their samples, things would be a lot easier. But then again, documentation is demanding and eats into tight time budgets for research (Bowker, 2008, p. 116), especially when you are a PhD student with a fixed contract and limited time to get things done. The opposite, however, is also true: Discerning the identity and ownership of poorly documented samples means just as much work, although work that is offloaded to others. To deal with the things that have amassed in the freezer, the Cardiff researchers have developed a number of routines and memory techniques over the years. Researchers are assigned to one or two specific freezers and their names are listed on a sheet of paper on the freezer door. Once a week, the group holds a lab meeting to discuss, update and remind members of what is going on; malfunctioning freezers and orphaned samples are a recurring topic. As is best practice in laboratories, people also keep lab journals in which they write down the experiments they have conducted as well as the samples they have used and produced (Roubert, 2017; see Breithoff & Harrison, 2020, p. 46 for an example). To prevent these documented memories from disappearing, students have to hand over their journals when they leave the lab. In addition, there is also a master spreadsheet table accessible from a dedicated computer in the lab, listing the boxes containing the samples, their location in the archive freezers, their contents, owners, investigators, research groups, contact persons and expiry dates (after which they can be thrown away). Despite all these precautionary measures, things are regularly forgotten by the diverse collective of researchers, labels, journals and excel sheets. What if the label says ‘John’ but there has not been a John working in the lab for some time? How does a label with a researcher’s name on it help with finding out whether a sample has survived thawing? What use is the information ‘June’ provided by someone who does not even work in the lab?

Mafalda’s problems with finding and identifying samples are thus not so much the result of poor documentation than of diverging documentations. Parts of the memories necessary for rendering samples intelligible and useful cannot be found in or on envelopes, on fridges and freezers or in spreadsheets. Instead, they still reside in the heads of absent researchers, in documentation that exists but is not readily connected to the samples and their containers, in deductions easily made in one place (the ‘June’ sample may be from a single-year project) but not in another (once the sample has sedimented into a cupboard spanning a decade of research). The containers that house the Cardiff samples—envelopes, bags, tubes, boxes—leave limited space for writing down information about their content and its origin (Breithoff & Harrison, 2020, pp. 43–47). The surplus of context, meanings and descriptions needs to be stored elsewhere, in different form. Rejoining the various elements of scientific memory is not straightforward. Next to information about the contents, samples should also be inscribed with pointers to places where additional information can be found: the names and mail addresses of collectors, an ID that reappears in a database, another freezer, a lab journal. These other places keep on moving, however, and as a result the pointers all too often become empty signifiers. This is especially true when references point to collectors and previous researchers who may have had an organized map of their collections in the past but with increasing spatial and temporal distance to the laboratory lose the ability to reconnect all the loose ends.

The various parts and members of the Cardiff laboratory move at different speeds and in different directions. Some people only stay for a few months and then leave, taking part of their knowledge with them. Others remain a part of the group for decades and can recite who was working with whom and on what materials. As a rule of thumb, samples tend to stay around for longer than people, although they tend to lose scientific value over time due to degradation of contents and informational references. The gap between a researcher’s stay and the lifetime of their samples is usually small enough to be bridged with enough effort, however. Someone somewhere will have an up-to-date mail address, a phone number, a vivid memory of what was happening three, five, or ten years ago. People may have left the lab but many of them have only moved to an adjacent one, or at least stayed in academia or in the Cardiff area. But what if the spatial scale is increased to all of the UK? This is an important question, since CryoArks seeks to connect hundreds of labs like the one in Cardiff to its federated infrastructure, meaning that a multitude of local memory systems will have to be linked to a central database. And what if the temporal gap between storage and retrieval widens to a time span greater than a researcher’s career, as is one of the key expectations behind CryoArks? What if storage temperature drops further, down to −80 or even −196°C? In an animal biobank, many of the issues that appear in the laboratory are amplified and reformulated, as the next section shows.

## The units of memory: Lessons from the cryobank

The NHM’s biobank in London is one of the key facilities of CryoArks. The NHM is part of the federated model of CryoArks, in which samples are stored in different locations across the UK but form part of the same larger collection. The NHM’s ‘molecular collection’, however, is also meant to take in outside material donated by third parties to store it in-house. In early 2020, together with Mafalda and a group of project students from Cardiff and Nottingham, I take part in a workshop led by Kirsty, the cryobank’s then technician. She has mailed us a best-practice manual for conducting a biobank inventory she has written in the run-up to the workshop (Lloyd, 2020), in which we are supposed to learn to transfer the contents of lab fridges and freezers to the CryoArks collection. Surrounded by rows of industrial freezers that can store samples at −80°C, Kirsty introduces us to the basics of animal cryobanking.

**Figure 2.**
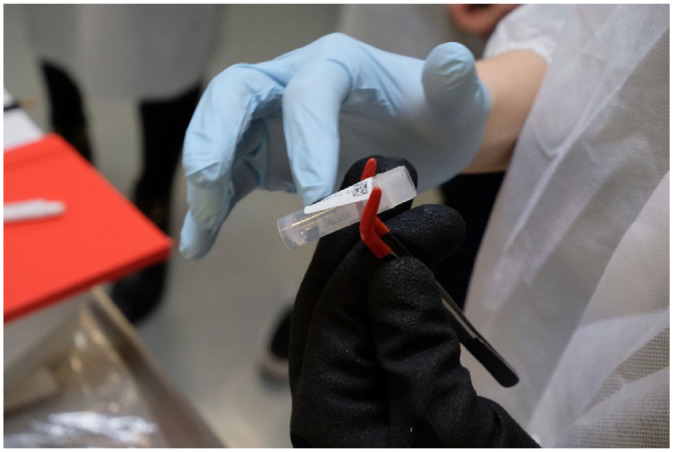
Labelling cryo-vials.

In accordance with Daston’s (2012) characterization of the ‘sciences of the archive’ as simultaneously backward- and forward-looking, the manual mixes retrospective and anticipatory perspectives. For example, it asks the user archaeological questions such as ‘What information do you have on the samples?’, ‘Where are they?’ and ‘Who owns them?’ about her collection, before prompting her to label and format them and enter the data into spreadsheets. This second, anticipatory process is also at the heart of the workshop. Robed in lab coats and gloves, we are supposed to go through the steps of ‘accessioning’, that is, the transformation of mere samples into standardized accessions, as biobankers call the items in their collection. On the top of a trolley-cart, utensils and equipment are prepared for us. Kirsty takes a pair of tweezers from the cart and uses it to retrieve a tiny plastic tube, known as a cryo-vial, from a plastic cassette.^4^ The task is to hold the tweezers with the cryo-vial in one hand while sticking a label onto them with the other (Figure 2). This is more difficult to do than it sounds or than it looks when Kirsty does it. Wearing gloves complicates the whole exercise even more. These labels come with a serial number and a barcode, which can be read by a scanner. Once the labels are attached, the students scan their vials, which allows them to enter the sample’s specifics into a software mask. After the data have been entered, the vials are put into another plastic cassette in a red plastic tub full of dry ice to keep them cool before they are ultimately stored in a −80°C freezer or liquid nitrogen.

The whole exercise is, of course, a dry run. No actual samples are used that day; all the vials are empty. But they do not have to contain samples for the process to work. What matters is that labels are attached to vials and those labels are scanned and linked to a database. Unlike the samples in the Cardiff lab, the freezers and cryotanks of the NHM cryobank cannot simply be opened and browsed for samples and information: The contents have to be stored at constant temperatures, so any unnecessary retrieval needs to be avoided. In an interview, Jackie, the Molecular Collection Facility’s manager and Kirsty’s then superior, explains the challenge as follows:

And using barcodes, the barcode postcode, we’d locate the actual position in one of our 24 -20 freezers, 17 −80 freezers, three liquid nitrogen [tanks], a slot of two million, exactly where that is. So you have to have, the location has to be very, very well established and direct, so that our museum curator can go and collect 30, 40, 50 samples, whatever they want, very quickly, and get them off the freezer without disrupting the rest of the content in the freezer.

Here is a first paradox of the biobank: Although it renders biological samples accessible in the long term (Lemke, 2023; Wolff, 2021), its cryogenic mode of storage also blocks easy access. Cryo-objects are thus available and unavailable at the same time. This obstacle can only be overcome with careful planning and targeted retrieval. If you want to get something from the biobank, you need to know where and what it is beforehand. The unavailability of samples is not only owed to the considerable thermal gap between their temperature and that of the laboratory. Even if they could be retrieved as easily as Mafalda’s envelopes from the cupboard, identifying their contents would largely be impossible. While plastic bags and paper envelopes come with enough surface to write down at least the most basic information—sex, date, location, species, breeder, fur color—the tiny surface of a cryo-vial barely leaves enough space to note down a species’ scientific name and glue a sticker to it. Neither do the contents give much clue about the identity of a sample. Blood, cells, or DNA can only be assigned with great scientific effort to an animal body or species. Unlike in the Cardiff lab, there is thus limited room for archaeological methods in the London nitrogen tank: What is forgotten cannot easily be recovered by other means.

## The cryo-object and its halves

To overcome the unavailability of samples, someone, or rather something, needs to remember their contents and locations. This is why the inscriptions attached to the stored samples, especially the adhesive labels, are so crucial for their longevity, not just as biological matter but as useful data from the past. To know which primer to use on frozen DNA or which technique to apply to old and dry hair follicles, researchers need to know the *contents* as well as the *contexts* of samples. In informatics and archival science (not to be confused with Daston’s sciences of the archive), the latter is expressed in metadata, that is, ‘data about data’ (Bowker, 2008, p. 116; Edwards et al., 2011). Metadata help users of archives locate single items in space and time, for example by assigning a book to a library shelf or an animal specimen to a particular person and a year of collection. It is through these metadata, together with her embodied memories, that Mafalda can correctly identify contents as ferrets rather than polecats or recognize other people’s samples as trout tissue. Mafalda’s envelopes and plastic bags can accommodate a fair number of such inscriptions, although they often suffer from a lack of standardization, such as when collaborators do not include a precise date or their handwriting is idiosyncratic. Envelope storage space, however, is still limited, not so much in terms of volume (many more hairs would fit in an envelope) as in terms of surface. And while plastic bags could in theory be wiped and relabeled, paper envelopes cannot. Excess memories, especially those that require recurrent updates or are needed to locate the physical sample in the first place, thus have to be stored somewhere else. The default location for this is inside the researchers’ heads—alas, such memories tend to default more often than is desirable.

Samples thus always have to be split up into data and metadata to be stored and remembered. Data usually require other modes of storage than metadata: The problem with blood is that it spoils and dries while the problem with lab books is that they are compiled in horrible handwriting. In certain contexts, data and metadata can nevertheless be stored side by side, for example in Mafalda’s cupboard or in the taxidermic collections of natural history museums, where labels with extensive information are attached to the preserved bodies of birds, mammals or insects (Van Allen, 2018). Once samples retreat into industrial freezers and cryotanks, however, data and metadata definitely need to be split in order to carry over into the future. The medium of the cryo-vial is crucial in this context: Its volume and surface area determine the maximum amount of information that can accompany biological matter into cold storage. A typical cryo-vial offers 1.8 ml for content and 10 cm² for context—any sample larger than that will have to be physically cut up, while any information requiring more space has to go elsewhere.

Two historical developments have made it easier for the life sciences to tackle this problem. The trend towards DNA and cells as the units of epistemic analysis (Keller, 2005; Landecker, 2007) today allows researchers to fill one cryotank with several thousand samples. Much more often than whole animal bodies (Van Allen, 2018), representative fragments such as ear biopsies, hair follicles, blood or single insect legs are filled into cryo-vials as a future source for molecular and cellular information. Cryopreservation of living matter could be understood as a ‘return’ to the cell as the smallest unit of biology (Landecker, 2007). In a wider sense, however, it also continues a trend away from bigger objects, as they are used in comparative zoology, physiology or ecology. Biobanks can only remember a particular size class of cryo-objects, which they in turn make proliferate. Aside from this size-specificity, however, the cryo-vial is surprisingly flexible. It can equally hold DNA, cells or blood, from mammals, insects or cephalopods, as long as they fit into the tiny space provided by its plastic walls and survive freezing and thawing. No matter how big or small animals may be, the size of their cells and DNA usually falls within the same order of magnitude. The appeal of cryobanks across the life sciences is that they promise to be of equal use to every sub-field and group of organisms.

More or less concurrent with the molecular and cellular developments in the life sciences, the advent of informatics and computers (Edwards et al., 2011; Hine, 2008; Nadim, 2021) has reduced the need for extensive paper documentation. As a result, Kirsty and her colleagues do not have to rely on labels bigger than their plastic vials. Metadata—that is, anything beyond organic snippets—is stored in computer systems such as the NHM’s in-house inventory database. Although Kirsty encourages us to write at least the species name on the tube, the bulk of memory work is thus taken over by computers and hard drives. The resulting arrangement resembles the distribution of memory in the Cardiff lab but differs in a few crucial aspects.

First of all, there are only two standardized storage systems, one for data and one for metadata, rather than several dozen different ones. The advantage of cryopreservation and informatics infrastructure is that as long as things come in the right form, cryo-vials and hard drives are indifferent to their contents. Blood, DNA, hair, feces, cells and so on can all be stored in the same containers at the same temperature. Similarly, their metadata, once digitized, can rest on the same drives. This reduces issues of compatibility and intelligibility that lab researchers encounter on a daily basis. Secondly, humans are largely cut out of the picture. Since the biobank is meant to give use to samples beyond a single project or career, it cannot rely on the embodied memory of individual researchers anymore: As far as possible, knowledge thus has to be turned into explicit information, formatted in a standardized way and documented in a universally intelligible manner. For this reason, there is no more space for handwriting in the biobank, and as many tasks as possible should be carried out by machines. In the NHM’s case, this currently applies to the entry of new files in the database only, although, as articulated in an interview, the biobanking team also dreams of an automated retrieval of samples.

Finally, and perhaps most crucially, the biobank rearranges a set of relations between the various descriptors and pointers that is still largely flat, redundant and open in the research laboratory. Cryo-vials will only be inscribed with one primary pointer: the label carrying the barcode and the serial number. This sticker points away from the contents of the plastic tube toward the metadata resting in the museum’s database. A barcode scanner connected to a computer will automatically open a corresponding file in the NHM’s lab management software when it reads the code on a label. Next to the unique serial number, this file will contain information about the storage conditions of the cryo-vial: storage medium, temperature, history of freezing and thawing, a brief description of the contents, a specimen number and link to a corresponding entry in the NHM’s overall inventory. Above all, however, it will also contain the precise location of the vial in the biobank’s space. This feature allows Kirsty and her colleagues to use their memory infrastructure from the opposite direction, that is, locating and retrieving one particular frozen sample by moving from the museum’s digital inventory via the lab management software to a freezer, a shelf rack, a drawer, a box, a single cryo-vial. To this end, biobanks rely on a particular spatial matrix wherein the physical location of an accession, can be described as [freezer/tank]—[shelf rack]^5^—[drawer]—[box]—[vial], each part of which can be expressed by a number (or letter), which can be stored in the laboratory management system. Locating vials this way, the database mirrors the nested architecture of the freezer—or, rather, the freezer is constructed in a way that facilitates the memory structure of the database (Harrison, 2017, p. 86). If contents were stored the way they are in an ordinary laboratory freezer (or a kitchen fridge), the system would have trouble remembering their whereabouts.

Taken together, these differences in infrastructure and sample preparation structure give the operation of ‘recalling’ (Peres, 2016) a particular character in the biobank. There is no place for rummaging, no guessing, no checking back with others, no trying to remember in this design. Instead, samples have to be rendered as explicit, discrete and unambiguous as possible. If recalling stored samples in the laboratory can be described as tying loose ends back together, the equivalent operation in the biobank resembles the rejoining of an object that has been split in two: a data and a metadata half. They are stored in different locations, at different temperatures and in different material states. Both halves only make sense in combination. Without contextual information, even a perfectly conserved frozen sample remains unintelligible; without the biological contents, the digitized context remains empty.^6^ In the Cardiff laboratory, several bits and pieces of metadata are stored in different places and states; often in redundant states and places that allow their reconstruction. In the London biobank, the metadata half of the cryo-object is kept in one place, which makes reconnection both more straightforward and more precarious. There is usually no route other than the barcode label in which a database entry and a sample can be linked again: only through scanning the barcode on the cryo-vial surface a second time can the identity of its contents be affirmed. This makes cryo-vials and their surfaces a crucial point of passage. If labels detached during storage or if barcodes became unreadable, both halves of the cryo-object would be lost. While this has yet to happen to samples in the CryoArks collection, it has already occurred in animal biobanks elsewhere (Van Allen, 2017, p. 538).^7^

## Planning hindsight

Especially at the conception stage of the CryoArks project, planning for the future largely revolved around anticipating future uses and users of the cryopreserved samples. The challenge was to build a system that no longer relies on the ability of human individuals to recall a particular sample, thus covering for the shortcomings of documentation. A future user must be able to retrieve and understand the contents and contexts of the freezers and tanks without assistance. Since most of the samples already have a specific format when they arrive at the NHM, they thus need to be reformatted, as Kirsty explains:

So you have to curate this, these collections so that they’re able to be loaned out, and that’s not just reformatting the samples themselves into appropriate long term storage tubes, like cryo-vials. That’s also dealing with the data. And if you get a collection that’s in a different state than you might expect or might want from a long-term perspective, and also you’re getting data from researchers that’s very specific to their own projects, but they might not have taken into account certain things like the factors that impact sample quality. So you need to aggregate that data altogether, make it into a format that is actually accessible, which means uploading it to some kind of database, into our management systems, so we know what everything is in the freezers.

Containers, inscriptions and documentation that originate in a lab context like the Cardiff one have to be migrated to a storage standard compatible with the London environment. This is a little easier on the cold end of things, where contents usually need to be moved from one plastic tube to another—provided sample quality, size and storage history allow for such a fiddly operation, which usually takes place at room temperature.^8^

The trickier part is to come up with a description that will still be useful and intelligible in 50 or 100 years: What would a future researcher need to know in order to put a sample to use? This undertaking is complicated by the diversity of zoological sub-disciplines, from population biology to zoo conservation to livestock breeding. For example, various fields need different resolutions of taxonomic information: A cattle breeder or a zoo veterinarian would be interested in the name of a breed or a pedigree, whereas a field biologist might content herself with a species name. Digital systems come with the advantage of vast storage space, so Kirsty, Jackie, Mafalda and their collaborators can include all of the existing documentation of a sample in the database. The downside of a unified system like the CryoArks database, however, is that every type of data requires a designated field: ‘*Gallus*’ goes in the genus field, ‘*gallus*’ goes in the species field, ‘*domesticus*’ in the subspecies field, for example. There is currently (2023) no possibility to search fields for breed information on the CryoArks database, however, which limits the group of future users to those zoologists whose taxonomies stop at the subspecies level.^9^

The conscious or unconscious inclusion of a group of future users and uses therefore entails more than just including information about a sample in the digital CryoArks database. The information itself needs to be reformatted and reorganized to fit into a matrix of fields in the front and back ends of the system. ‘[W]ith legacy collections themselves, they might not have it organized in the way that they can actually find things very easily, or the data might be a bit all over the place and you need to aggregate that together’, Kirsty explains. Even more crucially, a designated column will have to be created for each descriptor in advance. For example, it is difficult to create new columns within a running system for particular history of storage. The respective descriptors for older samples may already be included in other fields, were perhaps not included in the data migration, or cannot be retrieved retrospectively. The best point of time for data entry is when a sample goes into cold storage; revisions are often impossible for the same reason that rummaging around in a freezer does not work in a biobank. Planning hindsight in a biobank is thus more than envisioning a specific use for one particular sample: It implies imagining the full spectrum of possible uses for all samples in the frozen collection and constructing the database of the biobank accordingly. Difficulties arise both from the diversity of past storage conditions of the included samples and from the impossibility to foresee the requirements of future research techniques.

The physical formatting of samples exhibits a similar problem: Here, too, the biobank’s operators need to decide how their cryo-objects will be used in the future and subsequently inscribed in the physical matrix of the freezers and tanks. In this calculation, the CryoArks staff have to estimate how valuable a sample is, how much of it will be needed for a research project, and how many ‘duplicates’ they should accordingly preserve for the future: A unique, scientifically valuable specimen will likely be split across many tubes while conversely, fewer samples will be taken from individuals less sought after in the future. Since ‘recalling’ samples for research purposes from the biobank is always destructive—used samples cannot be sent back and re-frozen—Jackie and Kirsty need to decide in the present how often a particular sample will be requested in the future and split it across a respective number of cryo-vials.

Although cryopreservation implies perfect storage, there is thus plenty of planning and maintenance work needed before biobanks can actually preserve material memories. Descriptors must be translated to a standardized format, samples have to be transferred from one container to another, from minus 20°C to liquid nitrogen. Information has to be separated into data (frozen samples) and metadata (contextual memories and locators). Central for the ‘hindsight’ part of ‘planning hindsight’ is the ability to rejoin the two halves, frozen and digitized, of the cryo-object upon retrieval. In the above account, this ability hinges first and foremost on the proper arrangement of technology: a spatial and a corresponding digital matrix, linked through the signifier of the barcode. The capacities to remember, reconstruct and recontextualize research data are largely relegated to an infrastructure that is meant to survive the bodies and minds of human individuals.

Planned hindsight thus should not be understood merely as an imaginary among researchers (Radin, 2015) but rather as a property of a finely tuned biobanking apparatus of containers, labels, databases, and other technical elements. If this apparatus is meant to make human memory redundant, its designers and technicians have to plan, that is, inscribe (Akrich, 1992) both their visions of the future and future users’ access to them into it. In this process, ‘planned’ gradually loses its connection to human minds and becomes a term for the rigid pathways of sample storage and retrieval that the biobank relies on. There is no place anymore for ‘unplanned’ memory work. Similarly, ‘hindsight’ is no longer just a way of re-narrating past motivations behind stockpiling biological material. In a much more profane sense, it becomes the ability to ‘look back’ into the biobank, to perform the operation of rejoining data and metadata halves of its cryo-objects. Unlike in the laboratory or other, more open archives like a museum’s taxidermic collection, the path for this is a determined one running through the structure of the digital database, the spatial layout of freezers and tanks, and barcoded labels as an obligatory link. Although this version of planned hindsight in many ways overlaps and intersects with Radin’s original formulation, it diverges from it in its drive to reduce potentiality and speculation, instead replacing them with more specific anticipation and prediction (Anderson, 2010): The fewer unknowns, the better.

## Conclusion

I have described the biobank as an *animal* biobank here, primarily by situating it between mundane zoological laboratory routines and the natural history museum with its extensive wet and dry collections of animals. This comparison can be useful to highlight the impact of biobanks on these contexts as well as the continuities and breaks between them. It is not meant to say, however, that there is necessarily something particularly zoological about animal biobanks. The more professional and standardized a biobank becomes, the more it resembles any other biobank: from the outside, it is impossible to say what the tanks, freezers, and vials harbour. Consequently, the only thing reminiscent of animals in the NHM’s molecular collection rooms are pictures of animals—a polar bear, an arctic fox, an arctic hare, etc.—on the freezer doors that bear no relation to their contents: they simply serve as short hands for the biobank’s staff when speaking about a particular freezer. Apart from such details, the technology and infrastructure used by CryoArks could as well be used in a medical context or for environmental samples.^10^ This is owed to a general trend towards cells and molecules as objects of analysis in the life sciences, which can be accommodated in the tiny cryo-vials while representing individuals, species, and populations. This trend becomes most apparent where it is difficult to follow, such as in plant science, otherwise a pioneer in the cold preservation of biological material (Curry, 2022). The difficulties to apply cryopreservation techniques to so-called ‘recalcitrant seeds’ (Wyse et al., 2018; also Panis et al., 2020) and the continuing reliance on macroscopic seed as a format in plant biobanks (Chacko, 2018, 2019) point to the particularity of this model.

In turn, situating the animal biobank vis-à-vis seed banks also reveals that several of its features might best be understood as more generally owed to widening gaps in temperature, space, and time. The use of barcodes as ‘immutable mobiles’ (Eisenstein, 1979; Latour, 1987), a nested system of containers and a splitting of accessions into physical contents and digitalized contexts can also be observed in seed banking (Alpsancar, 2016; Chacko, 2018; Harrison, 2017; Peres, 2016). Like the animal biobank, the seed bank is designed to remove embodied human knowledge and semiotic ambiguities from the equation, which echoes the logic of bureaucratization in organizations more generally (Brans & Rossbach, 1997; Luhmann, 1964). Nevertheless, I believe the animal biobank warrants treatment as a paradigmatic rather than particular form of scientific memory.^11^ It intensifies temperature and standardization, apparent in its juxtaposition to both the laboratory and the seed bank. The latter still know at least a small to medium spectrum of size classes and different materialities, testified by the diversity of storage systems and containers that can be found in seed banking to this day (Karafyllis & Lammers, 2017). Seed banks’ walk-in freezers are still partially open to inspection, and the use of ‘active’ samples as a proxy (Chacko, 2018, p. 177) allows seed banks to inspect accessions that remain unavailable in animal biobanks. The former are thus closer to archives of printed and written documents than the latter (Peres, 2016).

If the animal biobank appears closely modelled after human biobanks, it is not just because it too manages to cut its accessions down to tissue, cells, or molecules. It heavily relies on standards, consumables and technical devices originally designed for medical biobanks and benefits from the economies of scale that result from a growing market for these applications. At the same time, the consolidation of the cryotechnology industry has also led to a consolidation of storage systems and their media. Many alternatives to cryo-vials developed in the early days of cryopreservation (Anonymous, 1988; Jackson et al., 1981; Radin, 2017, p. 72f.; Taylor, 1972) only survive in early biobanks today, while the cryo-vial and its complementary environment of stickers, scanners, cassettes, racks, freezers and tanks have become the standard for more and more applications. If these were developed around human medical practices, zoology and veterinary science have managed to adopt them—although likely by remodelling their practices and objects to make them compliant with the infrastructure, rather than the other way around.

The victory march of the cryo-vial in animal biobanks and beyond warrants critical reflection. When samples are split between vials and datasets, might there be a third part that is lost in the process?^12^ This question is difficult to answer now. Loss of memory will become apparent only in the future, when both halves are rejoined and found to amount to less than the original sample. The continued existence of ‘backups’ of data and metadata in laboratories and seed banks, however, suggests that other institutions cannot or do not want to rely on only one rigid pathway of recall. Consequently, their scientific memory features alternative, redundant and mediating channels through which memories can circulate between past, present, and future. Kirsty’s insistence that we use the tiny surface of the cryo-vials to leave an additional note for the future, shows that CryoArks researchers are not oblivious to this fact—just like Mafalda’s request in early 2022 to help her carry an archive of two dozen lab books from Cardiff to London. The books, the CryoArks researchers hope, will provide a different way of recalling the identity of frozen cell samples acquired earlier if digitized metadata will not suffice. Some hindsight, Radin (2015, p. 372) remarks, cannot be planned, only prepared for. With planned foresight expanding in the form of a single standardized system of memory preservation, it will become all the more important to look for redundancy in and between the crammed spaces of freezers, tanks, and cryo-vials.

## Notes

1. This semantic link between ‘bank’ and ‘value’ is an obvious one and therefore readily (perhaps too readily) drawn (e.g. Breithoff & Harrison, 2020; Radin, 2015; Swanson, 2014). One of the problems of this connection is that it tends to frame value as either residing in bio-objects or in their future imaginations (Tyfield 2009; see also Liburkina, 2023, for a critical perspective).

2. Although zoological research biobanks different in purpose and materiality from a ‘frozen zoo’, many of the latter originated in the former. See, for example, Friese’s (2013, pp. 108–110) account of the San Diego Frozen Zoo.

3. The freezers and fridges in the lab are ordinary consumer-grade models that can also be found in private households. Readers will recognize many of the problems described in this part from their own freezers and fridges (Shove & Southerton, 2000), especially if they share them with other people: What’s in this frozen box? Whose yoghurt is this? The Cardiff laboratory has merely taken a few extra measures to overcome the limitations of more conventional in-freezer documentation, which predominantly relies on adhesive labels and permanent markers.

4. The cryo-vial or cryotube has become a standard medium in cryotechnological storage, even if its precise size and shape vary. It is engineered so as to survive great differences in temperature while protecting its contents from leaking into the tank or freezer and the cooling agent from mixing with the samples it contains.

5. Owing to their design, cryotanks do not have shelves. Tower racks are instead lowered vertically into the tank. The NHM numbers its freezer accessions from the top left of the uppermost shelf downwards.

6. Chacko (2018, p. 148) notes that the former insufficiency is also used deliberately by plant seed banks to prevent theft.

7. Bangham (2020, pp. 211–214) describes another such scenario in a vignette of a frozen blood collection originally stored at the NHM and subsequently transferred to Cambridge. Although the blood remains well-preserved and documentation and lab books were transferred together with it, the custodians of the collection increasingly struggle to reconstruct the link between individual vials and the accompanying records. The case predates digital technologies, but similar problems can easily emerge with a change of standards and devices.

8. For handling samples that come out of cold storage, Kirsty advises us to fill a polystyrene box with dry ice to bridge the temperature gradient and minimize thawing damage to samples.

9. This likely reflects the absence of livestock breeders and veterinarians in the CryoArks consortium.

10. Although the digital documentation will differ in these cases, it is thus not uncommon to find biobanking practitioners from vastly different life sciences at the same biobanking conferences and workshops—they usually gather around the technology and particular biobanking standards rather than the ultimate applications.

11. For a contrary opinion, see Karafyllis (2018).

12. For seed banks (van Dooren, 2007; von Verschuer, 2021), and to a lesser extent for animal biobanks (Breithoff & Harrison, 2020; Chrulew, 2017), commentators have pointed out the ontological limits of an *ex situ* approach to conservation of organisms and species. What I am interested in here, however, are the epistomologcal limits of scientific memory in biobanks, rather than the viability of cryogenic life.
